# Effects of non-invasive brain stimulation on emotion regulation in patients with attention deficit hyperactivity disorder: a systematic review

**DOI:** 10.3389/fpsyt.2024.1483753

**Published:** 2024-12-04

**Authors:** Fang Shen, Hui Zhou

**Affiliations:** Department of Pediatrics, West China Second University Hospital, Sichuan University, Key Laboratory of Birth Defects and Related Diseases of Women and Children of Ministry of Education (MOE), Chengdu, China

**Keywords:** attention deficit hyperactivity disorder, emotion regulation, transcranial direct current stimulation, repetitive transcranial magnetic stimulation, systematic review

## Abstract

**Background and objective:**

A growing body of research evidence suggests that many patients with attention deficit hyperactivity disorder (ADHD) have difficulties with emotion regulation. Non-invasive brain stimulation (NIBS), which mainly includes transcranial electrical stimulation (tES) and repetitive transcranial magnetic stimulation (rTMS), has been considered a potential new direction in the treatment of emotion dysregulation in ADHD patients. The key components of tES are transcranial direct current stimulation (tDCS) and transcranial alternating current stimulation (tACS). However, there is no systematic evaluation exploring the effects of non-invasive brain stimulation on emotion regulation in ADHD patients. Therefore, this systematic review aimed to summarize the effects of NIBS on emotion regulation in ADHD patients.

**Methods:**

This systematic review was conducted according to the Preferred Reporting Items for Systematic Reviews and Meta-Analyses (PRISMA) guidelines. We searched PubMed, Embase, Web of Science, and the Cochrane Library electronic databases up to 1 July 2024. We also hand-searched the reference lists of retrieved articles and reviews. Assessing risk of bias using the Cochrane Assessment Tool.

**Results:**

Through database search, we obtained a total of 1134 studies, of which 5 met the inclusion criteria. Statistically significant improvements in emotion regulation in children with ADHD were observed in 1 study after treatment with tDCS. In the remaining 4 studies (2 with tDCS and 2 with rTMS), there were no statistically significant changes in emotion regulation in ADHD patients after treatment with either tDCS or rTMS.

**Conclusions:**

The data from our preliminary study do not allow us to draw definitive conclusions that non-invasive brain stimulation improves emotion regulation in ADHD patients. This is because there is a paucity of literature on the effects of tES or rTMS on emotion regulation in ADHD patients and a limited number of randomized controlled trials. More high-quality multicenter randomized controlled trials exploring the efficacy of non-invasive brain stimulation on emotion regulation in ADHD patients are needed in the future to provide strong evidence for definitive conclusions before it can be considered as a potential treatment option.

**Systematic review registration:**

https://www.crd.york.ac.uk/PROSPERO/, identifier CRD42024569041.

## 1 Introduction

ADHD is a common neurodevelopmental disorder in childhood, which is characterized by inattention, hyperactivity and impulsivity, and its symptoms often extend into adulthood (1). The global prevalence of ADHD in children is as high as 7.2% (2, 3), and the latest prevalence of ADHD in China is 6.4% (4). Furthermore, the global prevalence of adults with ADHD is 2.58% (persistent disorder) and 6.76% (symptomatic disorder) (1). Research has shown that the core deficits of individuals with ADHD are cognitive impairments. Meanwhile, a growing number of studies have found that emotion dysregulation is also common in people with ADHD, with prevalence rates as high as 60% in clinical samples (5, 6). Emotion regulation is the ability of an individual to alter affective states to promote adaptive, goal-directed behaviors and is critical for adaptive functioning throughout development (7, 8). Emotional dysregulation involves several complex domains of context-generating processes, including emotion recognition/understanding (ERU), emotion reactivity/negativity/lability (ERNL), emotion regulation (EREG), and empathy/callous-unemotional traits (ECUT) (9).

ERU refers to a young person’s ability to process and infer the emotions of others as well as his or her own. Various research groups have created reliable and valid standardized tasks assessing youth’s ERU such as asking youth to name emotions presented in pictures of faces or video vignettes, or the use of a Prosody Test (10–12). ERNL refers to an individual’s threshold, intensity, and duration of emotional arousal. Measurement of ERNL can be captured via rating scales, such as the anger/frustration scale of the Children’s Behavior Questionnaire (13), the emotional lability subscale of the Conners’ Parent and Teachers Rating Scales (14) or the lability/negativity subscale of the Emotion Regulation Checklist (15). There is no single definition of EREG, from a top-down perspective, EREG refers to responding effectively to emotional reactivity in a flexible way that promotes adaptive functioning (16). EREG measurements include the regulation subscale of the Emotion Regulation Checklist (15), the emotion control subscale of the Behavior Rating Inventory of Executive Function (17) as well as observationally via coding of the youth’s effectiveness in maintaining interest in a frustrating/challenging task. The cognitive aspect of empathy typically refers to a person’s ability to understand another person’s affective or cognitive state, while the affective component refers to experiencing another person’s affective state and/or expressing concern for another person (18). Low levels of empathy, guilt, and caring for others have recently been categorized under the term callous-unemotional (CU) traits (19). CU measurements include the Antisocial Process Screening Device (20) or the Inventory of Callous–Unemotional Traits (21). **Table 1** shows the measurement of multiple domains of emotional dysregulation.

**Table 1:** Shows the measurement of multiple domains of emotional dysregulation.

The process of emotional dysregulation	Measures
emotion recognition/understanding (ERU)	name emotions presented in pictures of faces or video vignettes, or the use of a Prosody Test
emotion reactivity/negativity/lability (ERNL)	Children’s Behavior Questionnaire Conners’ Parent and Teachers Rating Scales Emotion Regulation Checklist
emotion regulation (EREG)	the regulation subscale of the Emotion Regulation Checklist the emotion control subscale of the Behavior Rating Inventory of Executive Function coding of the youth’s effectiveness in maintaining interest in a frustrating/challenging task
empathy/callous-unemotional traits (ECUT)	Antisocial Process Screening Device Inventory of Callous–Unemotional Traits

Emotional dysregulation includes emotional instability, poor frustration tolerance, and the presence of negative mood symptoms such as irritability, anxiety, and depression. In the literature on ADHD, mood dysregulation has been conceptualized as emotional charge, difficulty in trying to regulate induced emotions, and difficulty in triggering positive, more acceptable affective states (16, 22). In children with ADHD, persistent mood dysregulation in childhood predicts the development of mood disorders in adolescence and adulthood and leads to higher rates of psychiatric co-morbidity, greater impairment of social functioning, and more persistent ADHD symptoms (23–26). Therefore, effective treatment of mood dysregulation in ADHD patients has become a widespread concern.

Brain regions involved in emotion regulation include the prefrontal cortex (PFC) (especially the ventral medial prefrontal cortex (vmPFC)), the anterior cingulate cortex (ACC), the limbic system, the parietal lobe, and the neural networks surrounding these brain regions. Of these, the PFC plays an important role in the regulation and control of emotions (27, 28). It has been found that PFC activity is enhanced when individuals are emotionally regulated, whereas these brain regions are diminished (29) or overexcited (30) when individuals are emotionally dysregulated. Elevating the level of PFC activation through external means may be an effective way to improve an individual’s emotion regulation (31).

NIBS is clinically important in neuropsychiatric disorders, and its use in neurodevelopmental disorders, especially ADHD, is still in its early stages but shows good promise. The most commonly used NIBS techniques are tES and rTMS. TES includes tDCS and tACS. RTMS is a non-invasive brain stimulation technique, which can directly stimulate the brain tissue non-invasively. Among them, the emerging theta burst stimulation (TBS) is a patterned rTMS modality that mimics the release frequency of gyrus impulses in the human brain (32). Compared with conventional rTMS, it has the advantages of high stimulation frequency and short stimulation time. There are two common TBS paradigms: intermittent theta burst stimulation (iTBS) and continuous TBS (cTBS). iTBS has an excitatory effect on the stimulated brain area, while cTBS has an inhibitory effect on the stimulated brain area (33–35).

NIBS alters cortical excitability and the metabolic activity of neurons in stimulated areas in a manner that does not require surgical intervention. Evidence from physiology, pharmacology, and behavior suggests that the modulatory effects of NIBS may arise through plasticity mechanisms (36). TDCS and rTMS have been shown to induce long-term enhancement or long-term inhibition in stimulated brain regions (37, 38). It has been suggested that NIBS offers the opportunity to study brain function by modulating the excitability of target brain regions (39). TDCS delivers low-intensity direct current through electrodes connected to the scalp. The most common intensity of tDCS is 1-2 mA for 20-40 minutes. It is generally accepted that anodic stimulation increases cortical excitability; conversely, cathodic stimulation inhibits cortical excitability. TACS applies a weak electric current with a sine-wave pattern to the scalp. Thus, it can modulate cortical oscillations that mediate cognitive functions and selectively modulate oscillations at the applied frequency (40–42). RTMS uses a short, strong current pulse delivered to a coil to create an electric field in the brain by electromagnetic induction. High-frequency rTMS (≥5Hz) increases cortical excitability, while low-frequency rTMS (≤1Hz) decreases cortical excitability (36).

There have been numerous reviews summarizing brain imaging studies of emotion regulation (28, 29). The core neural network mechanisms are the PFC brain regions responsible for cognitive control functions (mainly including dorsolateral prefrontal cortex (dlPFC), ventrolateral prefrontal cortex (vlPFC), parietal lobe, and supplementary motor areas) and the brain regions responsible for emotional response functions (subcortical regions including amygdala, ventral striatum, and central gray matter of the midbrain, and cortical regions including the insula and the dorsal ACC). Since the PFC is the core triggering brain region in the neural circuit of emotion regulation, most of the NIBS-based emotion regulation interventions have targeted this region for stimulation. The idea that activation of the PFC by NIBS enhances emotion regulation has been expressed. However, researchers have suggested that NIBS not only alters the activation level of the PFC, but also modulates the activity of deeper brain regions, such as the ACC, insula, and amygdala (43). Recent studies have found that TMS stimulation of the vlPFC elicits enhanced amygdala activity. This suggests that targeting cortico-subcortical structural connections can enhance the effects of scalp TMS on subcortical neural activity (44). The above implies that NIBS not only affects stimulated targets, but also affects deeper brain regions and covariation between different brain regions to achieve emotion regulation and mood improvement (45). However, there is no systematic review aimed at summarizing the effects of NIBS on emotional dysregulation in patients with ADHD.

## 2 Methods

### 2.1 Search strategy

The results of this study were searched for literature by the Preferred Reporting Items for Systematic Reviews and Meta-Analyses (PRISMA) statement guidelines (46). The protocol was registered under the PROSPERO ID CRD42024569041. Systematic and computerized searches were completed in the following electronic databases (as of July 1, 2024): PubMed, Embase, Web of Science, and the Cochrane Library. The search keywords are listed below: (a) “non-invasive brain stimulation” or “transcranial direct current stimulation” or “tDCS” or “transcranial magnetic stimulation” or “tACS” or “transcranial alternating current stimulation” or “rTMS” or “transcranial electric stimulation” or “iTBS” or “intermittent theta burst stimulation” or “cTBS” or “continuous theta burst stimulation”; and (b) “attention deficit/hyperactivity disorder” or “ADHD” or “hyperkinetic disorder” or “inattention” or “hyperactivity” or “impulsivity”; and (c) “emotion dysregulation” or “emotion regulation” or “mood regulation” or “mood dysregulation” or “anger” or “affect dysregulation” or “irritability” or “frustration”. We also examined the reference lists of included studies to find other studies that were eligible for inclusion.

### 2.2 Eligibility criteria

The inclusion criteria used to select the studies were: (a) empirical studies with sufficient method details that applied tDCS, tACS, rTMS, iTBS or cTBS in children and/or adults with ADHD confirmed by either a clinical diagnosis (as defined by DSM/ICD criteria) or by meeting cut-off criteria for ADHD on validated ADHD scales; (b) the control group intervention is sham stimulation; (c) the study design is the inclusion of empirical papers of any type of design for statistical analysis, including cross-sectional, cohort, case-control studies, self-controlled before-and-after studies, and clinically randomized controlled trials published in peer-reviewed journals at any time from the date of the database until July 1, 2024; (d) the outcome indicator is any self- or third-party standardized measurement tool relating to emotion, affect, or mood regulation or emotional lability; (e) the search will be limited to studies published in English. The following exclusion criteria were applied: (a) case reports, conference abstracts, reviews, replications and non-English studies; (b) animal studies or *in vitro* studies; (c) studies with incorrect, missing or not easily extractable data information.

### 2.3 Studies selection

**Figure 1:**
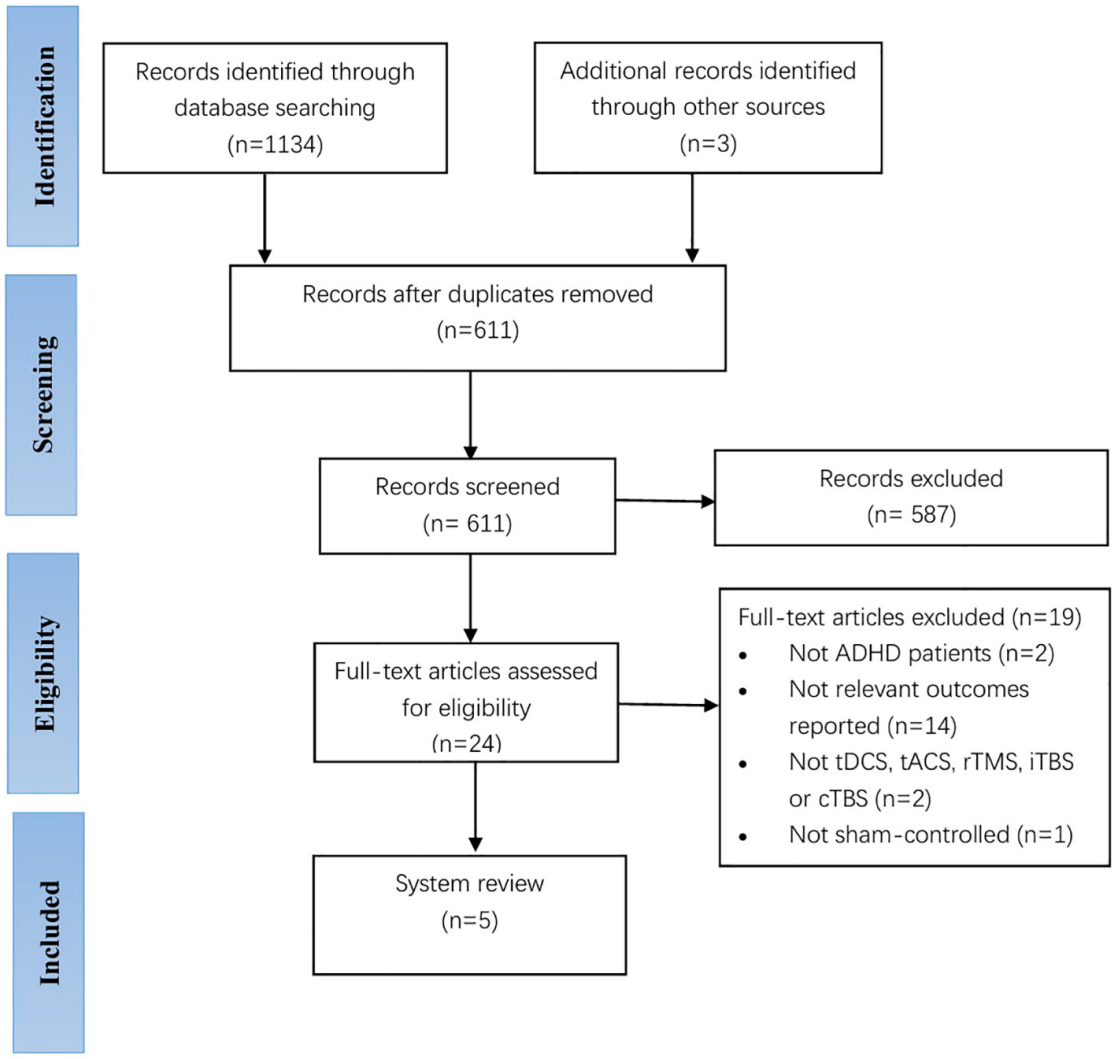
PRISMA flowchart reporting the screening process.

Two experienced researchers independently performed the search and selection of studies. A total of 1134 records were identified through database searching, and an additional three were identified after reviewing references of included manuscripts. We used EndNote V.X9 software to manage the literature and excluded 587 studies after removing duplicate 526 articles and then screening the titles and abstracts (47). After reading the full text of the remaining 24 articles, a total of 19 articles were excluded from further analysis, including 2 studies with no participants with an ADHD diagnosis, 14 studies with no change in mood symptoms in the outcome metrics, 2 studies with an experimental group that was not tDCS, tACS, rTMS, iTBS or cTBS, and 1 study with a control group with an intervention that was not sham stimulation. Finally, a total of five studies passed the selection criteria for the qualitative synthesis, including two studies for children with ADHD and three studies for adults with ADHD. TDCS had three studies and rTMS had two studies. **Figure 1** shows a flow chart of the studies from the systematic search to the selection process. Of note, we considered performing a meta-analysis but concluded that the results of the available papers were too varied to provide meaningful information for a meta-analysis.

### 2.4 Risk of bias assessment

The Cochrane Collaboration’s tool for assessing the risk of bias was applied to evaluate included trials in six domains - random sequence generation (selection bias), allocation concealment (selection bias), blinding of participants and personnel (performance bias), blinding of outcome assessment (detection bias), incomplete outcome data (attrition bias) and selective reporting (reporting bias) (48).

### 2.5 Data extraction

Two independent researchers performed data extraction. We extracted information on (a) study characteristics (e.g., authors, year of publication); (b) participant characteristics (e.g., age of participants, sample size); (c) study protocols including interventions and stimulus conditions (e.g., stimulus details including stimulus type, region, intensity, duration, and timing (online or offline); and (d) measurement methods and results (47).

## 3 Results

### 3.1 Characteristics of the studies

One author extracted data from the included studies for the scoping review. **Tables 2** and **3** summarize the characteristics and findings of the included clinical studies of tDCS and rTMS on mood dysregulation in patients with ADHD, respectively, all of which addressed the effects of tDCS or rTMS on mood regulation in patients with ADHD, including three tDCS, and two rTMS. The trials were published between 2010 and 2024 - one in 2024 (49), two in 2022 (50, 51), one in 2020 (52), and the other one in 2010 (53). And conducted in Iran (1 publication) (49), Turkey (1 trial) (50), and Israel (3 studies, one in collaboration with researchers from Israel and US) (51–53).

**Table 2:** Characteristics and findings of clinical studies included in the inclusion of tDCS for mood regulation in ADHD patients.

Authors	N	Mean age, year	tDCS Anode/cathode	Intensity	Duration, min	Time	Control	Stimulation sessions	Assessment tools	Effect
Estaji et al. (2024) (49)	24	9.09 ± 1.58 (range 6–12)	Left dlPFC/ Right vmPFC, Right vmPFC/ Left dlPFC	2mA	20min	Online	sham	1 session (*20 min) per week Total of 3 sessions (1 hours)	Emotional Go/No-Go task, Emotional 1-back task	Emotional Go/No-Go task (Go accuracy and RT [0]; No-Go accuracy [+]); Emotional 1-back (accuracy [+], RT [0]) (The effect of both electrode stimulation positions was consistent)
Barham et al. (2022) (50)	22	22.00 ± 2.77 (range 18–40)	Right dlPFC/ Left dlPFC	2mA	20min	Offline	sham	5 consecutive daily sessions (*20 min)	RMET	RMET scores [0]
Schertz et al. (2022) ‡ (51)	25	10.83 ± 1.79 (range 8–16)	Left dlPFC/ the scalp over the area of the vertex	1mA	20min	Offline	sham	3 sessions (*20 min) per week: Sunday, Tuesday, Thursday Total of 12 sessions (4 hours)	VADPRS, CBCL, BRIEF	VADPRS: Anxiety/depression scores [0], CBCL and BRIEF: Anxiety/depression, depression/seclusion and emotional control scores [0]

ADHD, attention deficit hyperactivity disorder; tDCS, transcranial direct current stimulation; dlPFC, dorsolateral prefrontal cortex; vmPFC, ventromedial prefrontal cortex; online, task performance during tDCS; offline, task performance after tDCS; RMET, the Reading the Mind in the Eyes Test; ‡, combined stimulation with cognitive training; *, refers to the time per session; VADPRS, Vanderbilt ADHD Parent Rating Scale; CBCL, Child Behavior Checklist; BRIEF, Behavior Rating Inventory of Executive Function; [+], statistically significant improvement; [0], not statistically significant.

**Table 3:** Characteristics and findings of clinical studies included in the inclusion of rTMS for mood regulation in ADHD patients.

Authors	N	Age, year	Region	Frequency, Hz	Intensity, % of RMT	Duration	Control	Stimulation sessions	Assessment tools	Effect
Alyagon et al. (2020) (52)	43	Sham: 27.64 ± 1.58 Active: 26.62 ± 0.66 (range 21–46)	Right PFC†	18	120	1440 pulses (2s on, 20s off)	sham	5 daily sessions per week for 3 weeks Total of 15 sessions	BDI	BDI scores [0]
Bloch et al. (2010) (53)	13	Not reported (adults)	Right dlPFC†	20	100	1680 pulses (2s on, 30s off)	sham	1 session per week Total of 2 sessions	PANAS VASs	mood and anxiety scores [0]

rTMS, repetitive transcranial magnetic stimulation; dlPFC, dorsolateral prefrontal cortex; RMT, Resting Motor Threshold; †, 5 cm forward to RMT point; BDI, Beck Depression Inventory; PANAS, Positive and Negative Affect Schedule; VASs, Visual analogue scales; [0], not statistically significant.

### 3.2 Risk of bias in studies

**Figure 2:**
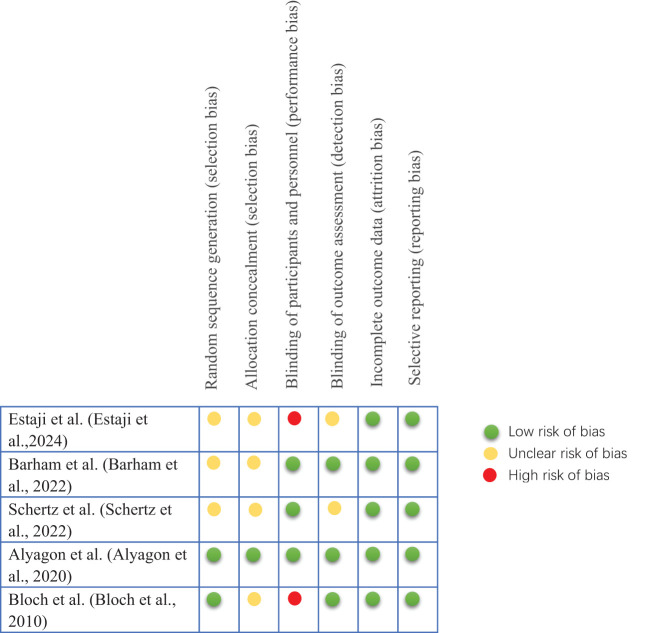
Risk of bias assessment.

The results of the included studies assessed using the Cochrane Collaboration’s Risk of Bias Assessment Tool are shown in **Figure 2**. Of the five manuscripts included in this systematic review, 40% had a high risk of performance bias. All trials had a low risk of lost visit bias and reporting bias. Studies with unclear risk of bias had higher proportions of allocation concealment (80%) and selection bias (60%) than other areas.

### 3.3 TDCS and emotional regulation in ADHD patients

In a single-blind, fully crossover design study, Estaji et al. (49) aimed to explore the effects of electrical stimulation of the left dlPFC and right vmPFC on emotion regulation in children with ADHD. Participants completed the Emotion Go/No-Go and Emotion 1-Back tasks five minutes after the onset of stimulation.

The Go/No-Go task is applied to evaluate pre-potent inhibition (54). In this task, participants were required to respond to the “Go” stimulus, but stopped responding if a “stop” signal appeared immediately after the “Go” stimulus. In the present study, the “Go” signal appeared within a frame that appeared in one of the four directions on the screen (right, left, up, and down) on each trial, and participants were asked to press the corresponding direction key as quickly and accurately as possible. On a few trials, an emotional picture appeared on the screen as a “No-Go” signal, in which case the subject had to refuse to respond. Thirty percent of the trials had a “No-Go” trajectory, which included an equal number of happy, sad, and neutral faces. In this task, “Go” accuracy and reaction time and “No Go” accuracy were outcome measures.

The Emotional 1-Back test was developed to measure emotion-related working memory performance (55). In this task, a sequence of stimuli is presented on a monitor and participants must judge whether each stimulus is identical or non-identical to the previous one. In this study, researcher randomly presented 100 facial images as stimuli and 30 identical images as responses. The target response images were happy, sad, and neutral faces, with 10 stimuli for each emotion. Each stimulus remained on the screen until a response was made. Accuracy and reaction time were outcome measures for this task and were indicators of working memory performance related to emotionally positive, negative, and neutral stimuli. Other details of the stimuli, including source, size, and presentation, were similar to the previous task (49). The results showed significant improvements in affective prioritization inhibition and affective working memory in children with ADHD in both real tDCS conditions. Furthermore, this study demonstrated that left dlPFC and right vmPFC are involved in emotion regulation in ADHD.

The remaining two randomized, double-blind, sham-controlled trials did not demonstrate the effectiveness of tDCS on emotion regulation in ADHD patients. In 2022, Barham et al. (50) used the RMET to assess face recognition, theory of mind, and emotion recognition skills (9, 56), and the results of the study showed no difference in performance on the RMET after tDCS treatment. However, this study found moderating effects of tDCS on planning and working memory in a small group of adults with ADHD.

In addition, Schertz et al. (51) randomly assigned 25 children with ADHD to receive 12 treatments, with the tDCS anode placed on the scalp overlying the dorsolateral prefrontal cortex area and the cathode placed on the scalp over the scalp over the area of the vertex. Outcome measures were taken before the intervention, and at two weeks (6 treatments), four weeks (12 treatments), and one month after the intervention, and total scores for anxiety/depression, depression/seclusion, and emotional control were measured using the VADPRS, CBCL, and BRIEF, and the study did not find statistically significant statistically significant results.

### 3.4 RTMS and emotional regulation in ADHD patients

In a semi-blind, randomized controlled trial of 43 medication-naïve adults with ADHD (52), patients received the stimulation location was the right dlPFC. The BDI was assessed using at pre-treatment, post-treatment, and follow-up, and the results of the study showed that dlPFC stimulation did not elicit changes in the BDI in this ADHD population.

In 2010, Bloch et al. (53) studied high-frequency rTMS of the right dlPFC in 13 ADHD patients in a crossover double-blind randomized, sham-controlled preliminary study. The assessment was conducted at the beginning of each day and 10 minutes after the administration of real/sham rTMS treatment, the scores for mood and anxiety were assessed using PANAS and VASs, and the results showed that there was no difference in the effect on mood, anxiety PANAS scores between post-real/sham rTMS compared to pre-real/sham rTMS. Meanwhile, the VAS score for mood showed no change in mood either real or sham rTMS.

## 4 Discussion

Studies have shown that approximately 25%-45% of children with ADHD and 30%-70% of adults with ADHD have difficulties with emotion regulation, which can lead to serious consequences (9, 16, 56, 57). These emotional difficulties have been described in a variety of ways (58). In the current study, a broad definition of emotion dysregulation was used, i.e., dysfunction in regulating affective states in an adaptive and goal-directed manner, resulting in individuals being easily agitated, rapidly angry, and experiencing strong mood swings (57). This may lead to excessive emotional expression and experience compared to social norms, as well as inappropriate emotional responses in specific situations. A growing body of research in recent years has focused on mood dysregulation in ADHD, with some researchers suggesting that mood dysregulation be considered a core feature of ADHD due to its prevalence in individuals with ADHD, its association with core ADHD symptoms, and its contribution to poor clinical outcomes (22, 56). Therefore, it is important to manage mood symptoms such as depression and anxiety in an optimal manner, especially mood dysregulation, which ultimately facilitates the recovery of higher-order dysfunctions such as cognitive and social impairments in ADHD patients (59). However, ADHD patients with mood dysregulation face significant treatment challenges, in part because clinical trials in ADHD have either not assessed changes in mood regulation or have treated it as a secondary outcome.

The current treatment modalities regarding mood disorders in ADHD patients are pharmacologic and nonpharmacologic. Evidence for psychostimulant efficacy on emotion dysregulation within ADHD is more limited. Medication for ADHD has been found to alleviate mood disorders, but the effects vary from study to study (58). Additionally, various side effects or adverse events during treatment with drugs for ADHD have been observed. Stimulants usually have adverse effects on sleep and appetite, and also cause side effects such as irritability, nausea/vomiting, abdominal pain, headache, mood swings, and growth inhibition, although these side effects are usually mild and may be temporary (60). More importantly, it is tolerated by only 50% of patients, caution is needed for certain co-morbid conditions (e.g., cardiovascular dysfunction and sleep problems), and adherence can be poor, especially in adolescence. Thus, the adverse effects and limitations of the medications limit treatment.

We conducted a systematic evaluation of NIBS on emotion regulation in ADHD patients, and based on the results of the data, few studies to date have investigated the use of NIBS in emotion dysregulation in ADHD patients. The trials included in this review addressed the effects of NIBS on anxiety, depression, emotion control, emotion recognition, emotion working memory, and emotion response inhibition in ADHD. The results of our systematic evaluation showed that for tDCS, 1 of the 3 included studies improved emotion regulation in ADHD patients after using tDCS stimulation with anodal dlPFC/cathodal vmPFC and anodal vmPFC/cathodal dlPFC, suggesting that dlPFC and vmPFC stimulation may be able to improve emotion regulation in ADHD patients (49). Of the three included studies on tDCS, only one observed a significant effect, whose outcome measure was a cognitive task measure with emotional stimuli, whereas the other studies had only questionnaire measures. In this one study where a significant effect was observed, a plausible explanation for the result is that cathodal tDCS in the right vmPFC reduces overactivity in this area, thereby reducing the likelihood that emotional stimuli will interrupt cognitive control. Furthermore, it can also be explained on the basis of emotional processing and its neurocognitive basis. Application of anodal tDCS to the left dlPFC reduces emotional attributions, makes emotional stimuli less salient, and facilitates cognitive control of emotional stimuli, resulting in more controlled responses in the affective versions of inhibitory control and working memory tasks. Thus, the significant effects on the Emotion Go/No-Go and Emotion 1-Back tasks may rely more on cognitive facilitation than on emotional processing facilitation. On the other hand, it may be that different types of measurements then (emotion-cognition tasks, questionnaires) are differently sensitive to stimuli (49, 61). But this finding needs to be confirmed by larger studies in the future. This study also demonstrated that dlPFC and vmPFC may be involved in emotion regulation in ADHD.

Nonetheless, 2 other studies, using tDCS to stimulate the anodal right dlPFC/cathodal left dlPFC, and the anodal left dlPFC/cathodal scalp over the area of the vertex, respectively, did not find that tDCS did not have an effect on emotional dysregulation in ADHD patients. Previous functional imaging studies have shown that the vmPFC and dlPFC interact with each other in emotion processing (62), specifically, the vmPFC is involved in attributing arousal to emotional stimuli (63), while the dlPFC is involved in evaluating the potency of that information (64). A review by Salehinejad et al. (65) also showed that emotion regulation involves the orbitofrontal cortex (OFC) and vmPFC. The 2 studies in our systematic review that did not find a clinical effect both involved stimulation of the dlPFC only and not the vmPFC, so further research is needed to demonstrate whether simultaneous stimulation of both the dlPFC and the vmPFC is required to improve emotion regulation in ADHD patients. Furthermore, in this study on tDCS improving mood dysregulation in patients with ADHD, the majority of participants had moderate ADHD, whereas in the other 2 studies with no significant improvement in mood dysregulation, the severity of the patients with ADHD was not specified, so further research is needed in the future to examine whether the baseline status of the patients (e.g., symptom severity) influences the NIBS on improving the mood dysregulation of ADHD patients’ results. In addition, previous studies have found that stimulating the same brain area (left dlPFC) with online and offline tDCS produced different results on emotion regulation (66, 67). Of the three studies on tDCS that we included, online tDCS was chosen for this study that improved mood dysregulation in patients with ADHD, whereas offline tDCS was chosen for the other two studies that did not produce an effect on the improvement of mood dysregulation. Therefore, online and offline tDCS may also be a potential factor influencing the results. Given the mixed results of NIBS interventions, more future studies are needed to validate the effectiveness and generalizability of NIBS for mood dysregulation in ADHD patients and to identify potential factors influencing intervention outcomes.

RTMS uses brief, intense pulses of electric current delivered to a coil placed on the subject’s head to create an electric field in the brain by electromagnetic induction. The effect depends on the intensity and duration of the stimulation; the number of stimulation pulses per second and their frequency, and the orientation of the coil. In general, based on exercise studies, high-frequency (>5 Hz) rTMS promotes cortical excitability, while low-frequency (1 Hz) rTMS inhibits cortical excitability (68). For rTMS, of the 2 studies included in this systematic evaluation, stimulation of the right PFC and the right dorsolateral prefrontal cortex using rTMS, respectively, did not reveal significant improvements in the emotion regulation dimensions in patients with ADHD, which may be related to the sample size, the intensity and duration of the excitation, the number of stimulation pulses per second and their frequency. There are few studies exploring the effects of rTMS on emotion regulation in ADHD patients to date, and more multi-session sham-controlled randomized trials with large sample sizes are needed to explore the effects of rTMS on emotion regulation in ADHD patients in the future in order to more thoroughly test the effects of rTMS under different protocols.

This systematic review is to summarize the effects of NIBS on emotion regulation in patients with ADHD, there are insufficient data to conclude that it improves emotion regulation in patients with ADHD, due to the small number of studies of NIBS in emotion regulation in patients with ADHD with small sample sizes, limited-quality experimental methodology, heterogeneity of protocols used, and a lack of long-term follow up to ensure that the efficacy is maintained, with only one study improving emotion regulation in patients with ADHD, and the results are not yet sufficient to draw conclusions about clinical benefit.

## 5 Future research directions

### 5.1 Reducing heterogeneity across studies

Current results on the effectiveness of NIBS intervention on emotion regulation in ADHD patients are inconsistent. This may be due to inter-study heterogeneity, such as individual differences in subjects, stimulus parameters (stimulus intensity, stimulus target, stimulus polarity, stimulus duration), and offline (NIBS conducted separately from the experimental task) versus online (NIBS conducted concurrently with the experimental task) NIBS, among other factors. The specific manifestations are as follows (1): Individual differences among subjects can lead to variations in experimental results, such as symptom severity, gender, age and subtypes of ADHD patients. Future studies would benefit by using more homogenous participants; (2) The selection of stimulation parameters (e.g., stimulation intensity, stimulation target, stimulation polarity, stimulation frequency and duration) may affect the effect of emotion regulation, and future studies need to systematically explore these parameters. Meanwhile, we can explore the precise regulation of emotion regulation in ADHD patients by multi-target NIBS, and observe the changes in the neural mechanisms under the joint action of multi-target NIBS by combining with functional magnetic resonance imaging (fMRI), electroencephalogram, and other brain observation techniques, so as to clarify the causal relationship of each brain region in the process of emotion regulation; (3) Both offline and online NIBS may influence the effectiveness of emotional regulation; (4) The sample size of the research program and the research methodology may also affect the effect of emotion regulation, and future studies with large sample sizes are needed to validate the effectiveness and generalizability of NIBS on emotion regulation in patients with ADHD, and to identify the potential factors that affect the results of the intervention, in order to realize better The results of this study are summarized as follows.

### 5.2 NIBS in combination with other treatments and application of multimodal NIBS

On the one hand, NIBS alters the excitability of the cerebral cortex and the metabolic activity of neurons in the stimulated area through electrode-induced electrical currents. It offers several advantages, including good tolerance, safety, non-invasiveness, and high compliance among children and their parents. NIBS can be used in combination with other treatment modalities, such as pharmacotherapy and psychotherapy. Future research needs to explore the potential and safety of combining NIBS with other treatments. Additionally, further studies are required to investigate whether the integration of NIBS with pharmacotherapy can reduce the dosage of medications while achieving optimal therapeutic effects. This approach may decrease the side effects of medications and enhance treatment compliance. On the other hand, multimodal NIBS, in which multiple NIBS techniques (e.g., rTMS and tDCS) are used concurrently, may be more effective than unimodal NIBS, and future research needs to explore the potential and safety of multimodal NIBS.

### 5.3 Neural network mechanisms of NIBS for emotion regulation in ADHD patients

Although research has shown that NIBS influences mood regulation in ADHD patients, its neural mechanisms are not yet fully understood. During the study of NIBS’s effect on mood regulation in ADHD patients, questions such as which deep brain regions are simultaneously affected when the PFC is activated by NIBS, and how the functional connectivity between the PFC and other brain regions changes, need further investigation. In the future, combining fMRI and functional near-infrared spectroscopy (fNIRS) could be used to explore the neural network mechanisms involved in mood regulation in ADHD patients under NIBS intervention.

### 5.4 Evaluating the effectiveness of the NIBS intervention on emotion regulation in children with ADHD from multiple dimensions

In many studies, the indicators reflecting mood regulation effects are primarily subjective experiences, namely self-assessment of emotional states by individuals and scores on emotion questionnaires. However, due to the influence of social desirability, subjects might deliberately conceal the true intensity of their subjective experiences, thereby affecting the study outcomes and failing to capture the full spectrum of the mood regulation process. Therefore, in future research, it is necessary to incorporate physiological measures (such as heart rate, blood pressure, pupil diameter, etc.) to explore the effects of mood regulation. Physiological indicators provide an unbiased, real response, and combining these with subjective reports can offer a more comprehensive evaluation of the intervention effects of NIBS on mood regulation in children with ADHD.

## 6 Conclusion

Based on the current research evidence, we are not yet able to recommend NIBS as alternative neurotherapies for the treatment of mood disorders in patients with ADHD. In addition, conclusive evidence from this systematic evaluation of NIBS studies is hampered by the heterogeneity of stimulation protocols, sample age, and mood outcome measures. Future larger, double-blind, randomized controlled trials using homogeneous protocols, systematically designed to be more optimal (e.g., multi-session interventions, different stimulation modalities, targeting different brain regions), and concurrently tested for mood-change outcomes are needed to assess their clinical efficacy and to provide clear guidelines for optimal stimulation protocols and stimulation of brain regions.
